# Artificial intelligence assisted colorectal lesion detection in private practices a randomized controlled study

**DOI:** 10.1038/s41746-026-02576-8

**Published:** 2026-04-01

**Authors:** Thomas J. Lux, Zita Saßmannshausen, Ioannis Kafetzis, Michael Banck, Adrian Krenzer, Daniel Fitting, Boban Sudarevic, Joel Troya, Wolfgang Boeck, Frank Passek, Tobias Heubach, Benjamin Simonis, Franz J. Heil, Leopold Ludwig, Frank Puppe, Wolfram G. Zoller, Alexander Meining, Alexander Hann

**Affiliations:** 1 Interventional and Experimental Endoscopy (InExEn), Internal Medicine II, University Hospital Würzburg, Würzburg, Germany; 2 Artificial Intelligence and Knowledge Systems, Institute for Computer Science, Julius-Maximilians-Universität Würzburg, Würzburg, Germany; 3 Gastroenterological practice Dres. Boeck/Haegele, Ulm, Germany; 4 Gastroenterological practice Bad Saulgau, Bad Saulgau, Germany; 5 Gastroenterological practice Heubach-Begetopoulos, Waiblingen, Germany; 6 Gastroenterological practice Darmstadt, Darmstadt, Germany; 7 Gastroenterological practice Andernach, Andernach, Germany; 8 Gastroenterological practice Dornstadt, Dornstadt, Germany; 9 Department of Internal Medicine and Gastroenterology, Katharinenhospital, Stuttgart, Germany

**Keywords:** Endoscopy, Colonoscopy, Cancer screening, Image processing

## Abstract

Computer-aided colonoscopy (CAC) may improve polyp detection and characterization compared to traditional colonoscopy (TC). However, recent studies also reported no relevant effect on adenoma detection rate (ADR). This study evaluates the real-time polyp detection system EndoMind during screening and surveillance colonoscopy in a multicenter randomized controlled trial. From November 2021 to November 2022, 933 individuals undergoing colorectal cancer screening or post-polypectomy surveillance were recruited and randomized in five outpatient treating centers (10 examiners; >10 years of experience). 914 Patients were included in the intention to treat analysis (CAC:452, TC:462) and detected lesions were framed on the primary monitor in the CAC group. More than 94% of the examinations were screening or surveillance colonoscopies with overall similar patient characteristics. The ADR (CAC:34.5% vs TC:32.9%; *p* = 0.656) was not significantly different between the groups. The effect of CAC on ADR remains a controversial discussion. Diverging study setups and patient collectives complicate consistent comparisons. Future studies should focus on large-scale real-world populations. ClinicalTrials.gov:NCT05006092 (registered: 2021-08-06).

## Introduction

Artificial intelligence (AI) has garnered significant attention in recent years, due to its potential to transform medical research and practice. Among the diverse areas of medicine where AI finds application, gastroenterology, specifically in colonoscopy procedures, is one that gathers increasing research interest.

Colonoscopy is a crucial tool for the early detection and treatment of precancerous and cancerous lesions in the colon^1^. Despite its importance, the procedure has limitations. Adenoma detection rate (ADR) - a key quality indicator of colonoscopy - can be influenced by factors including the skill of the endoscopist, quality of bowel preparation, and complexity of polyp morphology^2–4^. Missed lesions can lead to interval cancers^5,6^.

To mitigate these challenges, researchers are exploring the use of AI and deep learning algorithms to aid in the detection of polyps during colonoscopy, aiming to improve the ADR and reduce the adenoma miss rate (AMR). Studies investigating computer-aided detection (CADe) systems have shown promising results in enhancing polyp detection and ADR^7–12^.

Furthermore, the design and optimization of open-source CADe systems have been a focus of research^13,14^. While the utilization of AI in colonoscopy shows great potential and a multitude of positive RCTs have been published, it is important to note that its integration into clinical practice is still in its early stages^9,15^. Multiple prominent meta-studies provide a comprehensive overview of the use of CADe systems in colonoscopy procedures^16–18^. In many cases, ADR was significantly higher in patients who underwent colonoscopy with the help of CADe compared to those who underwent standard procedures. Sub-analyses confirmed the consistency of these results, including an examination of the first colonoscopy in tandem trials^19,20^. Interestingly, the benefits of CADe were more pronounced in studies with a low ADR compared to those with a higher ADR. However, many RCTs that focused on ADR with surveillance as main outcome reported a significant increase in ADR^20–22^. In contrast, latest research evaluating the effect of CADe on ADR after introduction into clinical practice and the usage of CADe in RCTs could not reproduce a significant ADR increase^23–27^. This was further demonstrated in a recent meta-analysis that presented a statistically but not necessarily clinically significant ADR increase due to the usage of CADe of less than 1%^28^. While first statements by societies have been published, the content remains controversial, and ranges “AI would be preferred by informed patients” to “AI should not be used” ^29–31^.

To further investigate the effect of computer-aided colonoscopy (CAC) in comparison to traditional colonoscopy (TC) in a multicentric, outpatient cohort including mainly screening patients, an RCT in multiple endoscopy private centers across Germany utilizing our developed CADe system, EndoMind, in clinical practice was performed. Our detection system is compatible with multiple endoscopic processors^11^ and achieves similar performance to that of commercially available CADe systems validated in RCTs, as was shown in a head-to-head comparison using a common benchmark dataset^32^.

## Results

### Study population

**Fig. 1:**
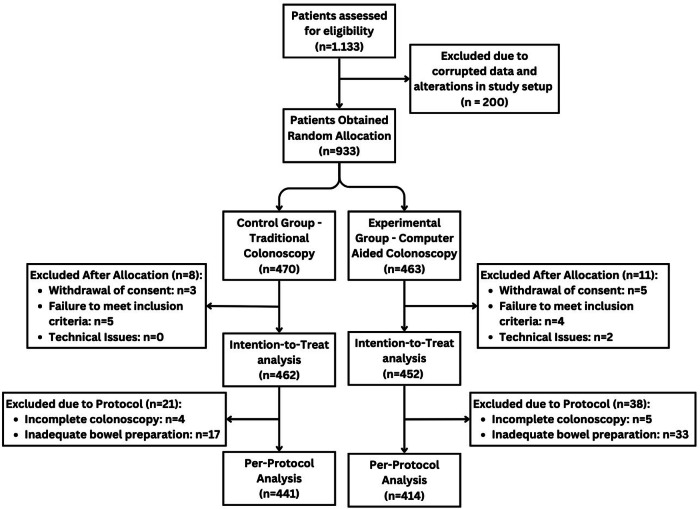
Consort flowchart of patient enrollment.

A total of 1133 patients were enrolled by 6 centers in the trial as planned in the sample size estimation. One center with 200 patients was excluded as explained in the methods section. Patient recruitment from November 5th 2021 to November 11th 2022 led to an initial total of 933 randomized participants. Of those, a total of 9 participants did not meet inclusion criteria, in 2 cases technical issues occurred, and 8 participants withdrew their consent resulting in a total of 914 patients for the intention to treat analysis (TC: 462, CAC: 452). Additionally, 50 individuals were excluded from the per-protocol analysis due to insufficient bowel preparation, and 9 due to an incomplete examination (Fig. 1). Following exclusions, 855 patients were incorporated into the per-protocol analysis (TC: 441, CAC: 414).

The patient characteristics for both TC and CAC groups were similar across most parameters, as seen in Table 1. The average age and gender distribution were similar in both groups, with a median age of 60 for the TC group and 61 for the CAC group, and females accounting for about slightly more than half (TC: 56.5%, CAC: 60.0%) of the participants. For the indication of the procedures, most cases were for screening purposes including post-polypectomy surveillance in both groups, with 94.2% in the traditional and 94.9% in the computer-assisted group (per protocol: TC—93.9%, CAC—94.4%). Patients with unclear indication and those with a positive iFOBT comprised the remaining cases. The used endoscope processors and BBPS (median, Q1-Q3: 7, 6-8) did not differ significantly between the two groups. Similarly, the self-reported patient characteristics BMI > 25 kg/m², alcohol consumption, tobacco use, red meat consumption, regular workout, usage of non-steroidal anti-inflammatory drugs or Acetylsalicylic acid, and family history of colorectal cancer, were also comparable between test groups allowing for a balanced comparison of the two colonoscopy methods (Supplementary Table 1).

**Table 1:** Patient and examination characteristics in the intention to treat analysis

Characteristic	Traditional colonoscopy	Computer-assisted colonoscopy
Number of participants	462	452
Age, median (Q1-Q3)	60 (56–67)	61(56–67)
Sex		
Female, n (%)	261 (56.5)	271 (60.0)
Male, n (%)	201 (43.5)	181 (40.0)
Indication		
Screening, n (%)	435 (94.2)	429 (94.9)
Positive iFOBT, n (%)	17 (3.7)	16 (3.5)
Not reported, n (%)	10 (2.2)	7 (1.5)
Endoscopic processor		
Olympus CV-190, n (%)	54 (11.7)	52 (11.5)
Olympus CV-170, n (%)	186 (40.3)	186 (41.2)
Pentax EPK i7000, n (%)	73 (15.8)	69 (15.3)
Storz Image 1S, n (%)	149 (32.3)	145 (32.1)
BBPS, median (Q1-Q3)	7.0 (6.0–8.0)	7.0 (6.0–8.0)

*SD* standard deviation, *iFOBT* immunochemical fecal occult blood test, *BBPS* Boston Bowel Preparation Scale, *Q1* first quartile, *Q3* third quartile.

### Polyp detection

The primary endpoint, the mean adenoma detection rate, was 32.9% in the TC compared to 34.5% in the CAC group (*p* = 0.656) and presented no statistically significant differences in the ITT analysis. Additionally, no statistically significant differences comparing the detection rates of polyps overall and serrated lesions were measured (Table 2).

**Table 2:** Detection rates of polyps, adenomas, and serrated lesions in the intention to treat analysis

Characteristic	Traditional colonoscopy	Computer-assisted colonoscopy	Difference in % (CI)	*p* value
ADR (%)	32.9	34.5	1.6 (–4.5 to 7.7)	0.6558
PDR (%)	54.5	53.8	–0.8 (–7.2 to 5.7)	0.8638
SDR (%)	9.3	7.5	–1.8 (–5.4 to 1.8)	0.3940
Mean number of adenoma per examination	0.57	0.58	0.01 (–0.13 to 0.14)	0.6035
Mean number of polyps per examination	1.59	1.50	–0.10 (–0.29 to 0.09)	0.6189
Mean number of SSL per examination	0.13	0.09	–0.04 (–0.10 to 0.01)	0.3092
Withdrawal time, median in minutes (Q1-Q3)	7.6 (5.6 – 11.7)	7.5 (5.8 - 10.4)	–0.16 (–0.78 to 0.45)	0.314
Withdrawal time without interventions, median in minutes (Q1-Q3)	6.5 (5.1–9.2)	6.6 (5.3–8.3)	0.09 (–0.37 to 0.53)	0.691
Procedure time				
AM, n (%)	299 (64.7)	290 (64.2)	–0.6 (–9.3 to 8.2)	0.9144
PM, n (%)	163 (35.3)	162 (35.8)	0.6 (–8.2 to 9.3)	0.9144

*ADR* adenoma detection rate, *PDR* polyp detection rate, *SDR* serrated lesion detection rate, *CI* 95% confidence Interval.

Considering the total of detected lesions in the ITT analysis, no significant differences in polyp sizes could be identified (Table 3).

**Table 3:** (**a**) Characteristics of detected lesions and (**b**) Characteristics of detected adenomas and (**c**) SSLs in the intention to treat analysis

Characteristic	Traditional Colonoscopy	Computer-assisted colonoscopy	Difference in % (CI)	*p* value
**(a) All Polyps**				
Total	517	465		
*(Location)*				**0.0078**
- Cecum, n (%)	67 (13.0)	41 (8.8)	–4.1 (–8.0 to -0.3)	**0.0489**
- Right Hemicolon, n (%)	218 (42.2)	173 (37.2)	–5.0 (–11.1 to 1.2)	0.1283
- Left Hemicolon, n (%)	159 (30.8)	188 (40.4)	9.7 (3.7 to 15.7)	0.0019
- Rectum, n (%)	73 (14.1)	63 (13.5)	–0.6 (–4.9 to 3.8)	0.8679
*(Size)*				0.3786
- <6 mm, n (%)	338 (65.4)	326 (70.1)	4.7 (–1.1 to 10.6)	
- 6–9 mm, n (%)	120 (23.2)	91 (19.6)	–3.6 (–8.8 to 1.5)	
- 10–20, n (%)	48 (9.3)	35 (7.5)	–1.8 (–5.2 to 1.7)	
- >20 mm, n (%)	6 (1.2)	9 (1.9)	0.8 (–0.8 to 2.3)	
- unknown, n (%)	5 (1.0)	4 (0.9)	–0.1 (–1.3 to 1.1)	
*(Shape)*				0.1612
- Pedunculated, n (%)	26 (5.0)	20 (4.3)	–0.7 (–3.4 to 1.9)	
- Sessile, n (%)	109 (21.1)	126 (27.1)	6.0 (0.7 to 11.4)	
- Flat, n (%)	83 (16.1)	74 (15.9)	–0.1 (–4.7 to 4.4)	
- unknown, n (%)	299 (57.8)	245 (52.7)	–5.1 (–11.4 to 1.1)	
*(Pathology)*				0.2114
- Non-neoplastic, n (%)	161 (31.1)	134 (28.8)	–2.3 (–8.1 to 3.4)	
- Tubular, n (%)	252 (48.7)	251 (54.0)	5.2 (–1.0 to 11.5)	
- Tubulovillous, n (%)	12 (2.3)	10 (2.2)	–0.2 (–2.0 to 1.7)	
- Sessile serrated lesion, n (%)	62 (12.0)	42 (9.0)	–3.0 (–6.8 to 0.9)	
- Malign, n (%)	1 (0.2)	5 (1.1)	0.9 (–0.1 to 1.9)	
- unknown, n (%)	29 (5.6)	23 (4.9)	–0.7 (–3.5 to 2.1)	
**(b) Adenomas**				
Total	264	261		
*(Pathology)*				0.8490
- Tubular, n (%)	252 (95.5)	251 (96.2)	0.7 (–2.7 to 4.1)	
- Tubulovillous, n (%)	12 (4.5)	10 (3.8)	–0.7 (–4.1 to 2.7)	
*(Location)*				0.1096
- Cecum, n (%)	30 (11.4)	22 (8.4)	–2.9 (–8.0 to 2.2)	
- Right Hemicolon, n (%)	130 (49.2)	111 (42.5)	–6.7 (–15.2 to 1.8)	
- Left Hemicolon, n (%)	82 (31.1)	107 (41.0)	9.9 (1.8 to 18.1)	
- Rectum, n (%)	22 (8.3)	21 (8.0)	–0.3 (–5.0 to 4.4)	
*(Size)*				**0.0303**
- < 6 mm, n (%)	162 (61.4)	193 (73.9)	12.6 (4.7 to 20.5)	**0.0028**
- 6–9 mm, n (%)	63 (23.9)	46 (17.6)	–6.2 (–13.2 to 0.7)	0.0980
- 10-20, n (%)	31 (11.7)	19 (7.3)	–4.5 (–9.5 to 0.5)	0.1112
- >20 mm, n (%)	4 (1.5)	1 (0.4)	–1.1 (–2.8 to 0.5)	0.3726
- unknown, n (%)	4 (1.5)	2 (0.8)	–0.7 (–2.6 to 1.1)	0.6857
*(Shape)*				0.1506
- Pedunculated, n (%)	22 (8.3)	15 (5.7)	–2.6 (–7.0 to 1.8)	
- Sessile, n (%)	60 (22.7)	81 (31.0)	8.3 (0.8 to 15.9)	
- Flat, n (%)	29 (11.0)	25 (9.6)	–1.4 (–6.6 to 3.8)	
- unknown, n (%)	153 (58.0)	140 (53.6)	–4.3 (–12.8 to 4.2)	
*(Dysplasia)*				0.9898
high grade dysplasia, n (%)	2 (0.8)	3 (1.1)	0.4 (–1.3 to 2.1)	
low grade dysplasia, n (%)	262 (99.2)	258 (98.9)	–0.4 (–2.1 to 1.3)	
Advanced Adenomas, n (%)	41 (15.5)	29 (11.1)	–4.4 (–10.2 to 1.4)	
**(c) Sessile Serrated Lesions**				
Total	62	42		
*(Location)*				0.3627
- Cecum, n (%)	14 (22.6)	6 (14.3)	–8.3 (–23.1 to 6.5)	
- Right Hemicolon, n (%)	45 (72.6)	31 (73.8)	1.2 (–16.1 to 18.6)	
- Left Hemicolon, n (%)	3 (4.8)	4 (9.5)	4.7 (–5.7 to 15.0)	
- Rectum, n (%)	0 (0.0)	1 (2.4)	2.4 (–2.2 to 7.0)	
*(Size)*				0.4649
- <6 mm, n (%)	27 (43.5)	18 (42.9)	–0.7 (–20.1 to 18.7)	
- 6–9 mm, n (%)	23 (37.1)	17 (40.5)	3.4 (–15.7 to 22.5)	
- 10-20, n (%)	11 (17.7)	4 (9.5)	–8.2 (–21.2 to 4.8)	
- >20 mm, n (%)	1 (1.6)	2 (4.8)	3.1 (–4.0 to 10.3)	
- unknown, n (%)	0 (0.0)	1 (2.4)	2.4 (–2.2 to 7.0)	
*(Shape)*				0.7033
- Pedunculated, n (%)	1 (1.6)	0 (0.0)	–1.6 (–4.7 to 1.5)	
- Sessile, n (%)	17 (27.4)	9 (21.4)	–6.0 (–22.6 to 10.7)	
- Flat, n (%)	30 (48.4)	21 (50.0)	1.6 (–18.0 to 21.2)	
- unknown, n (%)	14 (22.6)	12 (28.6)	6.0 (–11.2 to 23.2)	
*(Dysplasia)*				0.8800
- SSL with dysplasia, n (%)	3 (4.8)	3 (7.1)	2.3 (–7.1 to 11.7)	
- SSL without dysplasia, n (%)	51 (82.3)	34 (81.0)	–1.3 (–16.5 to 13.9)	
- unknown, n (%)	8 (12.9)	5 (11.9)	–1.0 (–13.9 to 11.9)	

Per protocol results were comparable and are illustrated in Supplementary Table 3a–c.

*CI* 95% confidence interval.

Regarding polyp morphology, the overall distribution was not different. When the frequencies for each shape were compared to the total, the percentage of sessile polyps tended to be higher in the CAC group (n.s.).

Finally, the location distribution of detected polyps shifted with the TC group having more detected polyps in the cecum (Difference –4.1%, 95-CI: –8 − –0.3, *p* = 0.049) and the CAC group having increased detections of polyps in the left hemicolon (Difference 9.7%, 95-CI: 3.7–15.7, *p* = 0.002).

Analysis of only the detected adenomas yielded similar tendencies regarding the location but without statistical significancy. Additionally, in the CAC group the fraction of small adenomas among the detected ones was higher (Difference 12.6%, 95-CI: 4.7–20.5, *p* = 0.0028). Correspondingly, the fraction of larger adenomas tended to be smaller in the CAC group; these differences were not statistically significant. In the PP analysis however, we observed statistically significant differences for the 6–9 mm (Difference -8.5%, 95-CI: –15.5 to –1.5, *p* = 0.0025) and 10–20 mm (Difference –5.5%, 95-CI: –10.5 to –0.6, *p* = 0.047) sized adenomas. Overall, similar results were obtained for the PP analysis and are reported in Supplementary Table 2 and Supplementary Table 3.

Lastly, we assigned examiners to “low” (*n* = 4), “medium” (*n* = 2), and “high” (*n* = 3) detector groups based on their ADR in the control group (low: < 25%, medium: 25–40%, high: >40%). One examiner contributing less than 5 examinations were excluded. Then, we compared differences between individual ADR with / without AI within those clusters. While the ADR difference of medium and high detectors was almost identical (Difference 0.9% [–12,6 to 14.3] and 0.9% [–25.7 to 27.4] respectively), the ADR of low-detectors tended to be higher with CADe support (Difference 5.9%, [–7.9 to 19.3], n.s.).

### Influence on withdrawal time

The median duration of the withdrawal phase of the examination was 7.6 min (Q1: 5.6; Q3: 11.7) for the TC group and 7.5 min (Q1: 5.8; Q3: 10.4) for the CAC group, presenting no significant difference. The corrected median withdrawal time was 6.5 minutes (Q1: 5.1; Q3: 9.2) for the TC group and 6.6 minutes (Q1: 5.3; Q3: 8.3), again presenting no significant difference.

## Discussion

**Fig. 2:**
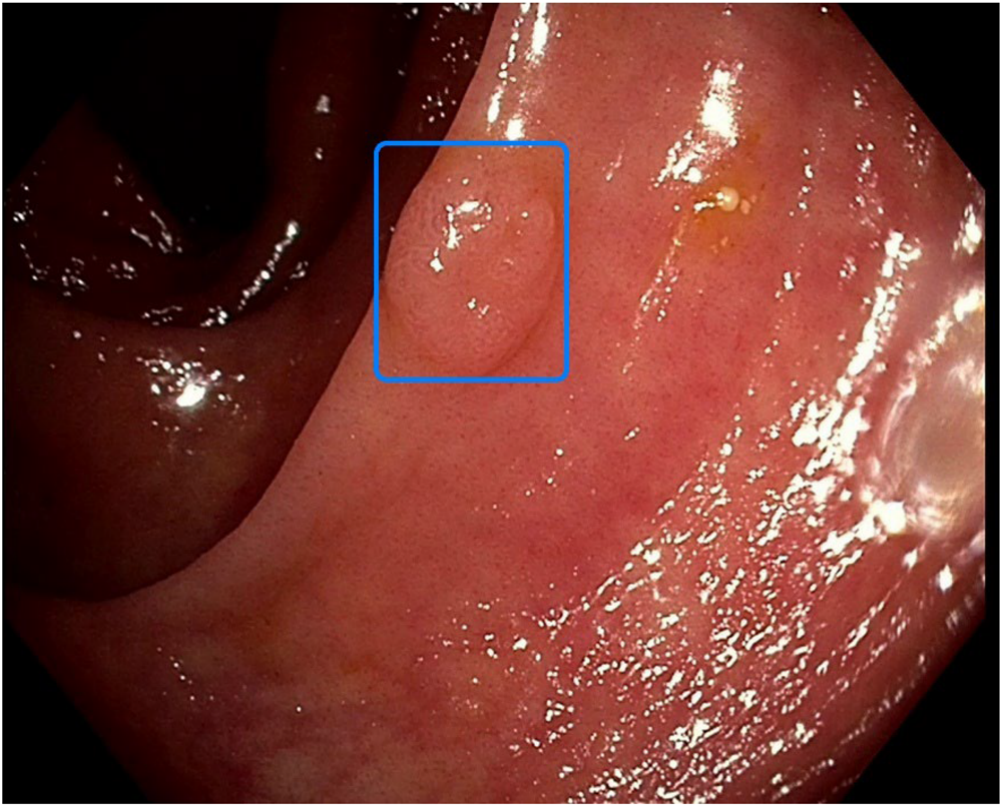
EndoMind system output. Example of the image displayed by EndoMind when an adenoma is detected. The blue square marks the adenoma.

This prospective, multicenter, randomized controlled trial assessed the potential advantage of utilizing a real-time polyp detection system in screening colonoscopy including post-polypectomy surveillance with a focus on outpatient treatment within a Western population (Fig. 2). Participating endoscopists were highly experienced and yielded a higher ADR in the control group than we assumed during the power calculation. In this context, we did not observe any additional benefit of computer-assistance regarding detection of adenomas during screening. ADR and PDR as well as the mean number of detected polyps/ adenomas per examination were similar among groups. When stratifying examiners by their ADR without AI support (low: <25%, medium: 25–40%, high: >40%), individual examiners ADR were most impacted by the CADe system in the “low” detector group (n.s.).

In contrast, previously published randomized controlled trials for the evaluation of CAC with ADR as a primary endpoint observed a mostly positive effect on polyp and adenoma detection rate with frequencies ranging from 25 to 61% for polyps and 8% to 44% for adenomas. For CAC the detection rates ranged from 39 to 67% for polyps and 16 to 55% for adenomas^7,20,22,33–42^. A major issue with comparability of those studies is the composition of the underlying study population which greatly influences the pre-test probability for lesion detection. While a recently published trial from Japan showed no significant difference (single, community-based center, *n* = 1041, ADR 54.5% (TC) vs 50.7% (CAC))^43^, a similarly designed trial from the United States focusing on a Hispanic population demonstrated a significantly higher ADR in the first half of the trial^44^.

To further illustrate potential contributing factors, we examined the methodology of previous RCTs. Of those studies, many were monocentric^7,20,33,35,37,38,40^. We also inspected where the study was performed and how the study population was recruited. Of the multicentric RCTs, few were performed on a western population (US^41^, Europe^34,36^). In those trials, the ADR of the TC group was 40–45%, and CADe increased the ADR by 4–14.5%, resulting in a detected adenoma in every second examination. Investigation of the patient cohorts revealed that the examinations’ indication for both European trials was “screening” in less than 40% of the cases.

Another large recent multicenter CADe RCT (US-based) revealed a significantly increased number of adenomas per colonoscopy while the ADR was not significantly different between CAC and TC^44^. Lastly, a recent comprehensive Meta-Analysis comparing 43 RCTs reported an overall higher ADR when applying CADe systems. Anyhow, the authors rated the certainty of evidence as “very low” due to serious risk of bias, serious inconsistency, and publication bias^45^. The metric ADR itself is closely associated with the underlying patient cohort as these examples may illustrate: One recent study reports an ADR of up to 60% containing only 6.7% screening colonoscopies^46^. Another detected large ADR improvements in “*Average-risk* 50-75-year-old individuals who underwent screening colonoscopy”^47^ while 13.8% of the population *had a positive fecal immunochemical test*. On the other hand, a large scale retrospective analysis of over 200,000 screening endoscopies (Austria; 2012 – 2018; 290 endoscopists) resulted in an overall mean ADR of 21.78%^48^. While a clear upward trend in adenoma detection may be attributed to improved imaging, the various reported baselines indicate a strong dependence on the exact examined population including various geographic, ethnic and lifestyle confounders. Since the number of patients with at least one adenoma is pre-determined by the recruited patient sample, a high baseline may limit the practically achievable ADR increase. We therefore conclude that absolute comparisons of detection rates should be made with utmost care. As concluded by Soleymanjahi et al., many AI studies deviate from the strict ADR definition (patients above the age of 50; first colonoscopy; no prior testing) inclusion criteria^45^.

After manual, video based annotation of the withdrawal time, we did not see an increase in the CAC group as also observed in previous studies^7,20,33,35,41^. Anyhow, a previous study demonstrated that both median withdrawal time and ADR significantly increased if examiners knew that their withdrawal time was measured^49^. This controverse finding suggests that the highly regulated setting of clinical trials may, by design, influence outcomes which depend on individual examiners’ performance. Taking this into account, we must assume that the individual examiners’ motivation to participate as a potential confounder. In this respect, we also noticed that most RCTs were performed in a highly controlled and academic setting. In comparison, our trial was focused on a non-academic, private-practice outpatient setting representing the majority of screening colonoscopies performed in Germany^50^.Based on a microsimulation by Halvorsen et al.^30^ the recommendations regarding CADe application in colorectal cancer prevention range between “Against the Usage” (British medical journal^51^), and “Weak Recommendation” (European Society of Gastrointestinal Endoscopy^29^). Therefore, the role of CADe in CRC prevention requires continued evaluation. Recent research also illustrated how the frequent usage of AI systems may impact individual skills and lead to “AI dependent” endoscopists^52^. To ensure evidence driven future of CADe systems, we should closely monitor the impact of all systems used in clinical practice, e.g. in the form of large registries.

Since self-developed CADe systems may underperform compared to industrial alternatives, we evaluated EndoMinds performance in a previous study. Here, we observed reliable and fast detection of polyps during real-time application compared to two commercially available CADe systems^11,32^.

The limitations of our study include that it was powered to detect ADR changes of 9 percent points based on the observed effects in the 4 pre-existing controlled trials ranging from 8.8% to 15.2%^33–35,37^. Anyhow, this study’s determined overall ADR of 32.9% (TC) was higher than anticipated based on previous large-scale real-world datasets primarily featuring a screening collective^50,53,54^. This significantly increases the risk of an underpowered trial with a falsely negative outcome. Additionally, we had to exclude one study center which resulted in a smaller dataset. Furthermore, the maximal achievable ADR is determined by the population. If an examiner already detects at least one adenoma without AI, additional benefits could be obscured. Re-estimation of sample size based on the effect of CADe on AI in our data indicated a required sample size of >6000 patients per group. Another recent polyp CADe trial which also did not show significant ADR differences between groups encountered a similar issue^55^. This emphasizes that future research should establish standardized examiner, patient and examination baseline characteristics, especially indications. This would be an important step to better understand the current inhomogeneity of trial results.

As patients were stratified by the center, dropout of one center did not cause group imbalance. Regarding both those factors, we deemed a prolonged recruitment phase to reach the initially calculated case number not feasible. Finally, the study was limited due to unretrievable information in the examination reports, caused mainly by the lack of standardized reports accompanying the examinations. This circumstance was because the study was carried out exclusively in a non-academic outpatient (private practice) setting.

Our RCT showed that the application of the CADe system EndoMind did not improve the ADR of experienced examiners in screening colonoscopies including post-polypectomy surveillance examinations. Similar results were obtained regarding the secondary outcomes, where no significant increase in either PDR or withdrawal time was observed. Yet, results of previously published studies show improvements in polyp detection, especially for small lesions.

## Methods

### Study design & population

This prospective, multicenter, RCT compared the ADR between TC and CAC. The study was conducted from November 2021 to November 2022 in 6 centers. In one center, erroneous documentation and alterations to the CADe system setup resulted in a mismatch of video and report data and inability to correctly match the data. Therefore, the center was excluded from further analysis. Hence, in the following, the analysis of 5 outpatient treating centers will be described. Adult patients with an indication for colorectal cancer screening, post-polypectomy surveillance endoscopy, or with a positive fecal immunochemical test (iFOBT) if aged ≥ 50 years, were eligible for participation. Indication was determined by the treating physician and based on the German colorectal cancer guideline^56^. Based on each examinations report, the indication was categorized into (1) screening or surveillance (“screening”), and (2) positive iFOBT. Exclusion criteria were suspected inflammatory bowel disease, familial polyposis syndrome, or previous radiation or resection of colon segments. Written informed consent was obtained from all participants prior to enrollment.

### Randomization

Recruited participants were randomized in a 1:1 ratio to undergo either CAC or TC (centrally generated in the university hospital Würzburg, sealed envelopes, stratified per center, randomization block size = 10). Randomization by previously distributed sealed envelopes occurred immediately before the examination. Study enrollment and group assignment were conducted by each center’s study team. Enrolled subjects were blinded to the result of their randomization while endoscopists were not. Examinations were recorded irrespective of the group assignment. For examinations of the CAC group, the predictions of the CADe system were also stored together with the examination recording. Pathologists who evaluated retrieved polyps were also blinded to the study group allocation. Both groups were assessed for characteristics such as gender, age, indication for colonoscopy, procedure time, type of sedation, and risk factors for colon polyps such as nutrition, nicotine abuse, body mass index (BMI), and family history of colonic adenoma or cancer.

### Intervention

Colonoscopies considered for the study were conducted in 5 outpatient treating centers (private practice) in Germany by 10 examiners, each with a minimum of 10 years of experience in colonoscopy and over 10,000 performed colonoscopies. Based on this experience, we assumed expertise even though individual examiners’ quality performance measures were not known. The endoscopy processors used in the different centers were Olympus CV-190, Olympus CV-170, Pentax EPK i7000 and Storz Image 1 S. All participants were prescribed a standard split-dose bowel preparation regimen. The CADe was in operation throughout the whole procedure for both groups. The colonoscopies for both the TC and CAC groups were performed using a single monitor. Only for the CAC group, polyp detections of the CADe system were displayed on the monitor. Detected polyps were handled according to best practices, decided by the endoscopist performing the examination.

### Real-time polyp detection system

The CADe system EndoMind includes a high-performance computer and video grabber card, compatible with diverse endoscopy processors as previously described^11^. The system comprises software for real-time data acquisition, display, and polyp detection. The framework minimizes video delay by processing video signals into frames and parallelizing the workflow into display, AI inference, and recording pipelines. The AI, built on the YOLO v4 architecture and was trained with over 506.000 manually annotated images, aims to predict a bounding box for any polyps present in the endoscopic images used as input. Data annotation was carried out by a physician, cross-verified by a board certified gastroenterologist. An example of the camera signal displayed by EndoMind upon detection of an adenoma is displayed in Fig. 2.

### Outcomes

The primary endpoint of the study is ADR, defined as the proportion of individuals who undergo a complete colonoscopy and are found to have at least one adenoma. Anyhow, colonoscopies with inadequate bowel preparation and incomplete colonoscopies were included in the intention to treat analysis. Secondary outcomes include the polyp detection rate (PDR), referring to the number of examinations with a minimum of one detected polyp. Further secondary outcomes were the withdrawal time, the resection time, defined as the total time spent on polyp resections, and the rate of advanced adenomas. Adenoma was defined as one of the following histological findings if not otherwise specified: tubular, tubulovillous or villous. Sessile serrated lesions were evaluated separately. An advanced adenoma was defined as a polyp with one of the following features: ≥1 cm as documented by the endoscopist, with villous architecture on histology or with high-grade dysplasia^56^. Number of polyps, polyp morphology, histology, and grade of bowel preparation were obtained from the examination report. Indication for colorectal cancer screening included also post-polypectomy surveillance endoscopies. Time spent for withdrawal and time spent on polyp resections were manually annotated by reviewing the examination’s video recording. Other factors influencing the prevalence of colorectal polyps like BMI, consumption of red meat or usage Acetylsalicylic were obtained via a questionnaire (Supplementary Table 1). For further subgroup analysis, examiners were stratified as low (< 25%), medium (25–40%) or high (> 40%) detectors based on individual ADR in the control group. Examiners with less than 5 examinations were excluded from the analysis.

### Determination of withdrawal and resection time

The video recordings were used to determine withdrawal and resection time for each examination including both groups TC and CAC. Start of withdrawal was defined as the last documented image of the ileum or, if not available, the timestamp of the last image showing the ileocecal valve. The first frame outside the patient’s body marked the end of the withdrawal. Examinations without cecal intubation were excluded from withdrawal time analyses. Resection times included the time spent inspecting a polyp, cleaning, resection, and treatment of post-resection mucosal defects, if any. In a second step, we determined the corrected withdrawal time by subtracting the resection times from the entire withdrawal time.

### Sample size estimation & statistical analysis

Sample size estimation was designed to detect at least 9% increased ADR, assuming a baseline ADR of 25% in the control group^50,53,54,57,58^ and a targeted ADR of 34% in the intervention group. To achieve a statistical power of 90% and a two-sided alpha level of 5%, a total of 1070 patients (535 per group) were required for the study. The sample size calculation employed a two-sided Z-test with unpooled variance. The overall participant enrollment goal was 1130 to allow for potential exclusions or dropouts.

The Intention-to-treat (ITT) analysis included all randomized patients fulfilling inclusion criteria and receiving colonoscopy using the installed EndoMind study setup. The per-protocol (PP) analysis included only examinations with successful cecal intubation and adequate bowel preparation (Boston Bowel Preparation Scale (BBPS) > 5).

### Statistical methods

All analyses were two-sided with α = 0.05. Intention-to-treat (ITT) and per-protocol (PP) populations were analyzed separately. Categorical endpoints (detection rates and polyp-level characteristics) were summarized as counts and percentages. Global group differences (AI vs No-AI) used Pearson’s chi-square test; Fisher’s exact test replaced chi-square whenever any expected cell count <5. For multi-category variables we conducted: (1) global test, (2) one-vs-rest contrasts, and (3) pairwise category vs category Fisher exact tests. Effect sizes for binary detection endpoints included risk difference (AI − control, Wald 95% CI; Wilson (Newcombe) CI computed for QC), risk ratio, and odds ratio.

Multiplicity control used Benjamini–Hochberg false discovery rate (FDR) adjustment within each predefined metric family for primary detection endpoints and subgroup interaction tests. For multi-category one-vs-rest and pairwise contrasts Holm’s step-down adjustment was applied within that family of comparisons.

Lesion burden (counts per exam) and time variables displayed median (IQR), mean, and proportion of exams with ≥1 event. Between-arm comparisons used the Mann–Whitney U test. Hodges–Lehmann median differences with bootstrap percentile 95% confidence intervals (2000 resamples) were reported. Withdrawal times (base, resection, corrected) were compared via Mann–Whitney U. Polyp-level analyses excluded lesions annotated as terminal ileum prior to categorization.

### Ethical considerations

This study received approval from the ethical committees responsible for each study center, including Ethik-Kommission Landesärztekammer Baden-Württemberg (F-2021–047), Ethik-Kommission Landesärztekammer Hessen (2021–2531), and Ethik-Kommission Landesärztekammer Rheinland-Pfalz (2021–15,955). In alignment with the Helsinki Declaration of 1964 and later versions, signed informed consent was obtained from each patient prior to participation. The trial was registered on ClinicalTrials.gov (August 6^th^, 2021), trial register number: NCT05006092.

## Supplementary information

Supplementary information

## Data Availability

As required by the study's ethics vote, the datasets generated and analyzed during the current study are not publicly available due to privacy concerns but are available from the corresponding author on reasonable request. The underlying code for this study and training/validation datasets are not publicly available for proprietary reasons.

## Code availability

The underlying code for this study and training/validation datasets are not publicly available for proprietary reasons.
