# Supplementary material for: Artificial intelligence assisted colorectal lesion detection in private practices a randomized controlled study
Source: NPJ Digit Med. 2026 Apr 1;9:284. doi: 10.1038/s41746-026-02576-8 (PMC13053717; doi:10.1038/s41746-026-02576-8)
Supplement: Supplementary file 1 — Supplementary information [file 41746_2026_2576_MOESM1_ESM.docx]

**Supplementary Data**

**Supplementary Table 1** Self-reported patient characteristics. Available for 389 in the traditional colonoscopy and in 366 cases for the computer-assisted colonoscopy group; BMI: body mass index, NSAID: non-steroidal anti-inﬂammatory drug, CRC: colorectal cancer.

| **Characteristic** | **Traditional colonoscopy** | **Computer-assisted colonoscopy** | **p-value** |
| --- | --- | --- | --- |
| BMI > 25 |  |  | 0.544 |
| Yes, n (%) | 119 (30.6) | 99 (27.1) | 0.321 |
| No, n (%) | 261 (67.1) | 257 (70.1) | 0.398 |
| Not reported, n (%) | 9 (2.3) | 10 (2.8) | 0.893 |
| Alcohol use (> 5x300ml beer or 200ml wine per week) | | | 0.938 |
| Yes, n (%) | 103 (26.5) | 101 (27.6) | 0.792 |
| No, n (%) | 283 (72.8) | 262 (71.6) | 0.782 |
| Not reported, n (%) | 3 (0.8) | 3 (0.8) | 1.000 |
| NSAIDs or Acetylsalicylic acid use | |  | 0.865 |
| Yes, n (%) | 58 (14.9) | 52 (14.2) | 0.865 |
| No, n (%) | 327 (84.1) | 311 (85.0) | 0.806 |
| Not reported, n (%) | 4 (1.0) | 3 (0.8) | 1.000 |
| Regular red meat consumption (more than twice a week) | | | 0.823 |
| Yes, n (%) | 108 (27.8) | 102 (27.4) | 1.000 |
| No, n (%) | 267 (71.0) | 261 (71.8) | 0.977 |
| Not reported, n (%) | 5 (1.3) | 3 (0.8) | 0.788 |
| Tobacco use |  |  | 0.752 |
| Yes, n (%) | 61 (15.7) | 52 (14.2) | 0.642 |
| No, n (%) | 326 (83.8) | 311 (85.0) | 0.733 |
| Not reported, n (%) | 2 (0.5) | 3 (0.8) | 0.945 |
| Family history of CRC (first-degree relatives) | | | 0.494 |
| Yes, n (%) | 79 (20.1) | 62 (16.2) | 0.274 |
| No, n (%) | 305 (78.6) | 299 (82.4) | 0.299 |
| Not reported, n (%) | 5 (1.3) | 5 (1.4) | 1.000 |
| Regular workout (physically active for 30-60 minutes per day) | | | 0.961 |
| Yes, n (%) | 299 (76.9) | 284 (77.7) | 0.879 |
| No, n (%) | 85 (21.8) | 77 (20.9) | 0.855 |
| Not reported, n (%) | 5 (1.3) | 5 (1.4) | 1.000 |

**Supplementary Table 2** Detection rates of polyps, adenomas, and serrated lesions in the per-protocol analysis; ADR: Adenoma detection rate, PDR: Polyp detection rate, SDR: Serrated lesion detection rate.

| **Characteristic** | **Traditional Colonoscopy** | **Computer-assisted colonoscopy** | **Difference in % (CI)** | **p-value** |
| --- | --- | --- | --- | --- |
| Detection Rates |  |  |  |  |
| - ADR (%) | 32.9 | 34.1 | 1.2 (-5.2-7.5) | 0.7700 |
| - PDR (%) | 54.9 | 53.1 | -1.7 (-8.4-4.9) | 0.6598 |
| - SDR (%) | 9.8 | 7.2 | -2.5 (-6.2-1.2) | 0.2352 |
| Mean Detections per Examination | |  |  |  |
| - Adenomas | 0.57 | 0.57 | -0.01 (-0.15-0.14) | 0.7146 |
| - Polyps | 1.14 | 1.02 | -0.12 (-0.31-0.06) | 0.4875 |
| - SSL | 0.14 | 0.09 | -0.05 (-0.11-0.01) | 0.1789 |
| Withdrawal Time, median in minutes [Q1-Q3] | | |  |  |
| - raw | 7.6 (5.6-11.7) | 7.5 (5.8-10.4) | -0.16 (-0.82-0.42) | 0.3142 |
| - without interventions | 6.5 (5.1-9.2) | 6.6 (5.3-8.3) | 0.09 (-0.37-0.52) | 0.6916 |
| Procedure time |  |  |  |  |
| - AM, n (%) | 287 (65.1) | 265 (64.0) | -1.1 (-10.1-8.0) | 0.7985 |
| - PM, n (%) | 154 (34.9) | 149 (36.0) | 1.1 (-8.0-10.1) | 0.7985 |

**Supplementary Table 3.** **(a)** Characteristics of detected lesions and **(b)** Characteristics of detected adenomas and **(c)** SSLs in the per-protocol analysis. Per protocol results were comparable and are illustrated in Supplementary Table 3 a - c. CI: 95% Confidence Interval.

| **Characteristic** | **Traditional Colonoscopy** | **Computer-assisted colonoscopy** | **Difference in % (CI)** | **p-value** |
| --- | --- | --- | --- | --- |
| **(a) All Polyps** |  |  |  |  |
| Total | 496 | 420 |  |  |
| *(Location)* |  |  |  | **0.0204** |
| - Cecum, n (%) | 67 (13.5) | 40 (9.5) | -4.0 (-8.1-0.1) | 0.0772 |
| - Right Hemicolon, n (%) | 206 (41.5) | 155 (36.9) | -4.6 (-11.0-1.7) | 0.1738 |
| - Left Hemicolon, n (%) | 152 (30.6) | 167 (39.8) | 9.1 (2.9-15.3) | **0.0049** |
| - Rectum, n (%) | 71 (14.3) | 58 (13.8) | -0.5 (-5.0-4.0) | 0.9016 |
| *(Size)* |  |  |  | 0.0941 |
| - < 6 mm, n (%) | 325 (65.5) | 308 (73.3) | 7.8 (1.9-13.8) |  |
| - 6 - 9 mm, n (%) | 114 (23.0) | 74 (17.6) | -5.4 (-10.6--0.2) |  |
| - 10-20, n (%) | 46 (9.3) | 29 (6.9) | -2.4 (-5.9-1.2) |  |
| - >20 mm, n (%) | 6 (1.2) | 7 (1.7) | 0.5 (-1.1-2.0) |  |
| - unknown, n (%) | 5 (1.0) | 2 (0.5) | -0.5 (-1.6-0.6) |  |
| *(Shape)* |  |  |  | 0.2643 |
| - Pedunculated, n (%) | 25 (5.0) | 16 (3.8) | -1.2 (-3.9-1.4) |  |
| - Sessile, n (%) | 106 (21.4) | 112 (26.7) | 5.3 (-0.3-10.9) |  |
| - Flat, n (%) | 81 (16.3) | 65 (15.5) | -0.9 (-5.6-3.9) |  |
| - unknown, n (%) | 284 (57.3) | 227 (54.0) | -3.2 (-9.7-3.2) |  |
| *(Pathology)* |  |  |  | 0.2808 |
| - Non-neoplastic, n (%) | 154 (31.0) | 124 (29.5) | -1.5 (-7.5-4.4) |  |
| - Tubular, n (%) | 239 (48.2) | 223 (53.1) | 4.9 (-1.6-11.4) |  |
| - Tubulovillous, n (%) | 12 (2.4) | 10 (2.4) | -0.0 (-2.0-2.0) |  |
| - Sessile serrated lesion, n (%) | 62 (12.5) | 38 (9.0) | -3.5 (-7.5-0.5) |  |
| - Malign, n (%) | 1 (0.2) | 4 (1.0) | 0.8 (-0.3-1.8) |  |
| - unknown, n (%) | 28 (5.6) | 21 (5.0) | -0.6 (-3.6-2.3) |  |
| **(b) Adenomas** |  |  |  |  |
| Total | 251 | 233 |  |  |
| (Pathology) |  |  |  | 0.9683 |
| - Tubular, n (%) | 239 (95.2) | 223 (95.7) | 0.5 (-3.2-4.2) |  |
| - Tubulovillous, n (%) | 12 (4.8) | 10 (4.3) | -0.5 (-4.2-3.2) |  |
| (Location) |  |  |  | 0.2782 |
| - Cecum, n (%) | 30 (12.0) | 21 (9.0) | -2.9 (-8.4-2.5) |  |
| - Right Hemicolon, n (%) | 120 (47.8) | 103 (44.2) | -3.6 (-12.5-5.3) |  |
| - Left Hemicolon, n (%) | 79 (31.5) | 92 (39.5) | 8.0 (-0.5-16.5) |  |
| - Rectum, n (%) | 22 (8.8) | 17 (7.3) | -1.5 (-6.3-3.4) |  |
| (Size) |  |  |  | **0.0008** |
| - < 6 mm, n (%) | 155 (61.8) | 183 (78.5) | 16.8 (8.8-24.8) | **0.0001** |
| - 6 - 9 mm, n (%) | 59 (23.5) | 35 (15.0) | -8.5 (-15.5--1.5) | **0.0249** |
| - 10-20, n (%) | 29 (11.6) | 14 (6.0) | -5.5 (-10.5--0.6) | **0.0474** |
| - >20 mm, n (%) | 4 (1.6) | 1 (0.4) | -1.2 (-2.9-0.6) | 0.3741 |
| - unknown, n (%) | 4 (1.6) | 0 (0.0) | -1.6 (-3.1--0.0) | 0.1245 |
| (Shape) |  |  |  | 0.1902 |
| - Pedunculated, n (%) | 21 (8.4) | 11 (4.7) | -3.6 (-8.0-0.7) |  |
| - Sessile, n (%) | 57 (22.7) | 68 (29.2) | 6.5 (-1.3-14.3) |  |
| - Flat, n (%) | 28 (11.2) | 22 (9.4) | -1.7 (-7.1-3.7) |  |
| - unknown, n (%) | 145 (57.8) | 132 (56.7) | -1.1 (-9.9-7.7) |  |
| (Dysplasia) |  |  |  | 1.0000 |
| high grade dysplasia, n (%) | 1 (0.4) | 1 (0.4) | 0.0 (-1.1-1.2) |  |
| low grade dysplasia, n (%) | 250 (99.6) | 232 (99.6) | -0.0 (-1.2-1.1) |  |
| Advanced Adenomas, n (%) | 39 (15.5) | 22 (9.4) | -6.1 (-11.9--0.2) |  |
| **(c) Sessile Serrated Lesions** |  |  |  |  |
| Total | 62 | 38 |  |  |
| (Location) |  |  |  | 0.4671 |
| - Cecum, n (%) | 14 (22.6) | 6 (15.8) | -6.8 (-22.4-8.8) |  |
| - Right Hemicolon, n (%) | 45 (72.6) | 28 (73.7) | 1.1 (-16.8-19.0) |  |
| - Left Hemicolon, n (%) | 3 (4.8) | 3 (7.9) | 3.1 (-7.0-13.2) |  |
| - Rectum, n (%) | 0 (0.0) | 1 (2.6) | 2.6 (-2.5-7.7) |  |
| (Size) |  |  |  | 0.6243 |
| - < 6 mm, n (%) | 27 (43.5) | 17 (44.7) | 1.2 (-18.9-21.2) |  |
| - 6 - 9 mm, n (%) | 23 (37.1) | 15 (39.5) | 2.4 (-17.3-22.0) |  |
| - 10-20, n (%) | 11 (17.7) | 4 (10.5) | -7.2 (-20.8-6.4) |  |
| - >20 mm, n (%) | 1 (1.6) | 1 (2.6) | 1.0 (-5.0-7.0) |  |
| - unknown, n (%) | 0 (0.0) | 1 (2.6) | 2.6 (-2.5-7.7) |  |
| (Shape) |  |  |  | 0.8281 |
| - Pedunculated, n (%) | 1 (1.6) | 0 (0.0) | -1.6 (-4.7-1.5) |  |
| - Sessile, n (%) | 17 (27.4) | 9 (23.7) | -3.7 (-21.2-13.8) | |
| - Flat, n (%) | 30 (48.4) | 19 (50.0) | 1.6 (-18.6-21.8) |  |
| - unknown, n (%) | 14 (22.6) | 10 (26.3) | 3.7 (-13.7-21.2) |  |
| (Dysplasia) |  |  |  | 0.8188 |
| - SSL with dysplasia, n (%) | 3 (4.8) | 3 (7.9) | 3.1 (-7.0-13.2) |  |
| - SSL without dysplasia, n (%) | 51 (82.3) | 30 (78.9) | -3.3 (-19.4-12.8) | |
| - unknown, n (%) | 8 (12.9) | 5 (13.2) | 0.3 (-13.4-13.9) |  |
